# Editorial: Neuronal Stochastic Variability: Influences on Spiking Dynamics and Network Activity

**DOI:** 10.3389/fncom.2016.00038

**Published:** 2016-04-21

**Authors:** Mark D. McDonnell, Joshua H. Goldwyn, Benjamin Lindner

**Affiliations:** ^1^Computational and Theoretical Neuroscience Laboratory, Institute for Telecommunications Research, University of South AustraliaMawson Lakes, SA, Australia; ^2^Department of Mathematics, Ohio State UniversityColumbus, OH, USA; ^3^Theory of Complex Systems and Neurophysics, Bernstein Center for Computational NeuroscienceBerlin, Germany; ^4^Department of Physics, Humboldt University BerlinBerlin, Germany

**Keywords:** balanced network, channel noise, heterogeneity, Hodgkin-Huxley model, neural networks, neuronal variability, stochastic dynamics

Stochastic variability is present across all scales of brain activity. At the single-cell level, for instance, synaptic transmission is mediated by stochastic release of neurotransmitter and membrane potentials fluctuate due to random conformational changes of ion channels. When these cell-level sources of stochastic variability emerge at the network level, they generate fluctuating currents that drive complex network dynamics. Even if intrinsic cellular noise sources are neglected, the interaction of many nonlinear units in recurrent networks typically leads to an effective network noise which is often mathematically tractable in a stochastic framework.

This Research Topic brings together works that address the pressing challenges of developing computational tools and mathematical theories that advance our understanding of stochastic neural dynamics. Six contributions cover stochastic variability at the single-cell level. Moezzi et al. study synaptic coupling between inner hair cells and auditory nerve fibers. Three works update our understanding of ion channel noise in stochastic versions of the Hodgkin-Huxley equations (O'Donnell and Van Rossum; Pezo et al.; Rowat and Greenwood). Puzerey and Galán quantify information transmission in a stochastic Hodgkin-Huxley neuron model that receives barrages of balanced excitatory and inhibitory inputs. Lazar and Zhou communicate a modeling framework that includes dendritic processing of noisy inputs and channel-noise influenced spike generation.

The remaining four studies offer new perspectives on network dynamics. Dummer et al. works out the requirements for self-consistent input/output statistics for neurons embedded in recurrent networks. Lagzi and Rotter develop a Markov chain model that clarifies the stochastic dynamics of balanced networks. Mejias and Longtin explore effects of neural heterogeneity on network response properties. Lajoie et al. make elegant use of random dynamical systems theory to analyse stimulus encoding in in chaotic networks.

Two commentary articles are also part of this research topic: the commentary of Thomas on Lajoie et al. and the commentary of Baroni and Mazzoni on Mejias and Longtin.

## Stochastic variability in single neuron dynamics

Moezzi et al. study the sub-cellular origins of long and short-term correlations in inter-spike-intervals (ISIs) in spontaneously firing individual fibers of the auditory nerve, and the form of the distribution of ISIs. They hypothesize the existence of a pre-synaptic mechanism in auditory inner hair cells that causes randomly delays in the availability of synaptic vesicles immediately following prior release of a vesicle. They propose a model for this that produces a simulated ISI distribution equivalent to those in standard existing models. Building on this, the authors tackle the subcellular source of ISI correlations. They report that introducing a model of ion channel noise in the calcium channels of pre-synaptic inner hair cells, when combined with the random vesicle availability model, enables simulation results from the overall model to match the qualitiative nature of ISIs in auditory nerve fiber spontaneous spiking.

Rowat and Greenwood analyse the distributions of interspike intervals generated from simulations of stochastic Hodgkin-Huxley models using several available algorithms. They conclude that appropriate use of the diffusion approximation (e.g., Goldwyn and Shea-Brown, 2011) can yield accurate estimations of these distributions when compared to “micro-scale” simulations of Markov chain models of ion channel kinetics. The authors draw on their previous studies of Hodgkin-Huxley and related models (Baxendale and Greenwood, 2011; Rowat and Greenwood, 2011) to explain features of the computed interspike interval distributions including local maxima (“bumps”) and exponential shape in the tails of of these distributions.

Pezo et al. concur that diffusion approximations can provide accurate and efficient approximations to Markov chain models of channel noise. They caution, however, against using these methods for simulations of fewer than ~1000 channels. In this low channel number regime, Markov chain methods can be used without sacrificing speed or accuracy. Pezo et al. make the important point that membrane area is subdivided into compartments when simulating dynamics of spatially-extended neurons. The local channel count within each membrane compartment can be small, and thus Markov chain methods may be preferred in multi-compartment computations. Taken together, the Pezo et al. and Rowat and Greenwood articles provide a set of benchmark simulations that will guide researchers interested in studying channel noise in neurons.

O'Donnell and Van Rossum remind us that channel noise depends on the channel type. In stochastic versions of the Hodgkin-Huxley model, for instance, membrane fluctuations are driven by Na^+^ and K^+^ channels. Differences in the properties of these ion channels include the probabilities of being in the open states, the time scales of channels opening and closing, and the maximal conductances per channel. O'Donnell and Van Rossum introduce a method to quantify how each channel type contributes to membrane potential fluctuations and how these fluctuations trigger spontaneous action potentials. Importantly, their method can be applied to any channel type in a conductance-based model (as they illustrate with an analysis of a model of CA1 hippocampal neuron). As such, the methods presented by O'Donnell and Van Rossum should be essential to researchers seeking to estimate membrane potential fluctuations induced by diverse channel types.

Puzerey and Galán study responses of the stochastic Hodgkin-Huxley model to barrages of excitatory and inhibitory inputs. They quantify information transmission (measured from estimates of spike train entropy) for a range of synaptic time scales and delays between excitation and lagging inhibition. They find that synaptic kinetics modulate information transmission the most when synaptic currents are balanced and delays in inhibition are small (0.8 ms). The framework of these simulations (neuron model with channel noise, driven by balanced and noisy synaptic inputs) lays a foundation for future studies that will integrate sources of stochastic variability at the neuron and network levels.

Lazar and Zhou seek to identify the theoretical limits of precision with which noisy sensory neurons can encode and decode stimuli. To this end, they introduce an innovative abstract neuron model, comprised from a dendritic module and a spike-generating module, and study small circuits of such neuron models. The model exhibits a strong grounding in physiological detail; it includes active dendrites; it can use biophysical models such as the Hodgkin-Huxley model for spike generation; and most pertinently to this research topic, includes two intrinsic noise sources. The first source of noise is dendritic variability; the second is ion channel noise due to a finite number of channels (also the focus of O'Donnell and Van Rossum; Pezo et al.; Rowat and Greenwood in this topic). The authors provide extensive mathematical analysis and simulation results, and based on this argue that a duality between stimulus decoding and functional identification holds.

## Stochastic variability in neuron network dynamics

The study by Dummer et al. explores an important condition for the temporal correlations of spiking neurons within homogeneous recurrent networks. In such networks, the mean activity of every unit is determined in a self-consistent manner, i.e., its mean input (coming from similar neurons) is simply related to its mean output. Likewise, temporal input correlations are proportional to temporal output correlations and this fact can be used to determine the spike-train correlation function or, equivalently, the spike-train power spectrum, from iterative simulations of a single neuron. Dummer et al. compare two such iterative schemes to simulations of a sparse recurrent network and find excellent agreement. Moreover, their study proves that the emergent network noise can be strongly colored and that the shape of the power spectrum (the “noise color”) depends in a nontrivial way on cellular and network parameters.

Although single-cell spike train variability is an important proxy of network stochasticity, it does not necessarily give reliable estimates of the *multi-cell* spike-train variability that is important for encoding of time-dependent stimuli at the population level. Lajoie et al. study the multi-cell variability of a recurrent network of deterministic quadratic integrate-and-fire neurons (a highly chaotic system) under the influence of a set of frozen-noise stimuli. Results from the theory of random dynamical systems are used to estimate an upper bound of the noise entropy. This entropy quantifies the variability caused by different initial conditions of the chaotic system. Lajoie et al. show that, surprisingly, this upper bound of the multi-cell noise entropy is an order of magnitude lower than a naïve estimate that is based on the single cell's noise entropy. These results may pave the way for the estimation of the mutual information between multicellular spike patterns and complex spatio-temporal stimuli (see also the commentary article of Thomas).

A significant open question in understanding information processing and learning in mammalian cortex is whether the ubiquitous heterogeneity in the anatomy and physiology of cortical neurons and their synaptic connections are necessary for function, or makes little difference. Mejias and Longtin systematically investigate this question for a physiological parameter. They introduce heterogeneity into a standard population model of sparsely-connected excitatory and inhibitory cortical neurons that traditionally assumes homogeneity in all parameters. The heterogeneity in the model takes the form of random distributions for the membrane-potential threshold for action potential generation in individual neurons. The authors use simulations and mathematical analysis to explore the effects of heterogeneity, and report intriguing differences that result from whether it is excitatory neurons that are modeled as heterogeneous, or inhibitory neurons. Following publication, a commentary from Baroni and Mazzoni summarized the paper of Mejias and Longtin and discuss the many ways in which the work could be extended. They point out that different heterogeneities could interact in complex unpredictable ways, hence coining the phrase *heterogeneity of heterogeneities*.

Last but not least, Lagzi and Rotter also consider recurrently connected networks of excitatory and inhibitory neurons. They specifically consider the balanced-state of such networks, where population activity fluctuates but has a steady-state stable mean. Like Dummer et al. and Lajoie et al. the model considered has no external noise, and the fluctuations are a result of the underlying chaotic dynamics of the system. The central contribution of Lagzi and Rotter is to propose a two-state Markovian model which they show exhibits the same phenomenology and statistical properties as simulations of recurrently connected cortical networks in the balanced regime. The authors additionally derive corresponding mean-field equations and provide associated analysis.

## Outlook

The work collected in this Research Topic represents important advances in our understanding of stochastic variability of dynamics in neurons and networks. Methodological issues—such as preferred numerical methods for simulating channel noise in stochastic versions of Hodgkin-Huxley-type models—have been clarified. Moving forward, we expect to see more examples of models that are tightly constrained by known physiological data (as in Moezzi et al.). We have highlighted how variability, as interpreted in these works, can arise from stochastic cellular mechanisms (synapses, ion channels) and from nonlinear and chaotic network dynamics. Of great interest will be future studies that bridge these scales by exploring, for instance, how cell-level noise filters through populations of neurons to shape the dynamics and signal processing capabilities of neural networks.

## Author contributions

All authors listed have made substantial, direct and intellectual contribution to the work, and approved it for publication.

## Funding

MM's contribution was supported by an Australian Research Fellowship from the Australian Research Council (project number DP1093425).

## Acknowledgment

We thank all authors and reviewers of manuscripts submitted to this research topic.

## Conflict of interest statement

The authors declare that the research was conducted in the absence of any commercial or financial relationships that could be construed as a potential conflict of interest.
